# Molecular Cloning and Characterization of Full-Length cDNA of Calmodulin Gene from Pacific Oyster *Crassostrea gigas*

**DOI:** 10.1155/2016/5986519

**Published:** 2016-09-15

**Authors:** Xing-Xia Li, Wen-Chao Yu, Zhong-Qiang Cai, Cheng He, Na Wei, Xiao-Tong Wang, Xi-Qing Yue

**Affiliations:** ^1^College of Food Science, Shenyang Agricultural University, Shenyang 110866, China; ^2^School of Agriculture, Ludong University, Yantai 264025, China; ^3^Changdao Enhancement and Experiment Station, Chinese Academy of Fishery Sciences, Changdao 265800, China

## Abstract

The shell of the pearl oyster (*Pinctada fucata*) mainly comprises aragonite whereas that of the Pacific oyster (*Crassostrea gigas*) is mainly calcite, thereby suggesting the different mechanisms of shell formation between above two mollusks. Calmodulin (CaM) is an important gene for regulating the uptake, transport, and secretion of calcium during the process of shell formation in pearl oyster. It is interesting to characterize the CaM in oysters, which could facilitate the understanding of the different shell formation mechanisms among mollusks. We cloned the full-length cDNA of Pacific oyster CaM (cgCaM) and found that the cgCaM ORF encoded a peptide of 113 amino acids containing three EF-hand calcium-binding domains, its expression level was highest in the mantle, hinting that the cgCaM gene is probably involved in shell formation of Pacific oyster, and the common ancestor of Gastropoda and Bivalvia may possess at least three CaM genes. We also found that the numbers of some EF hand family members in highly calcified species were higher than those in lowly calcified species and the numbers of these motifs in oyster genome were the highest among the mollusk species with whole genome sequence, further hinting the correlation between CaM and biomineralization.

## 1. Introduction

Calmodulin (CaM) seems to play important roles in regulating the uptake, transport, and secretion of calcium in molluscan shell formation, such as yesso scallop (*Patinopecten yessoensis*) [1], freshwater pearl mussel (*Hyriopsis schlegelii*) [2], and pearl oyster (*Pinctada fucata*) [3]. The pearl oyster CaM protein or calmodulin-like protein (CaLP) was found to interact with different target proteins in the mantle and the gill [4] and may regulate the calcite growth in the shell prismatic layer by inducing the aragonite nucleation and modifying the calcite morphology in vitro crystallization experiments [5]. The pearl oyster shell mainly comprises aragonite [6, 7], whereas the Pacific oyster shell is mainly calcite [8]. The higher level of calcite in the oyster shell may suggest that CaM has a different function in the Pacific oyster. To elucidate the role of CaM in shell formation in the Pacific oyster, we cloned the full-length cDNA of Pacific oyster CaM (cgCaM) and analyzed its characteristics.

## 2. Materials and Methods

### 2.1. Specimens, RNA Isolation, and Primers

Six organ types (hemolymph, mantle, digestive gland, gill, adductor muscle, and labial palps) were collected from slaughtered Pacific oysters, snap-frozen in liquid nitrogen, and stored in the Ultra-low Temperature Freezer (−80°C) until extracting RNA. The total RNA was extracted using Trizol reagent (Tiangen, China). All of the primers were designed referring to one of the predicted CaM sequences (CGI_10006482) deposited in OysterBase (http://www.oysterdb.com/), as described in Table 1.

### 2.2. Cloning the Full-Length cDNA

Full-length cgCaM mRNA sequences were got by 5′-RACE and 3′-RACE. For the 5′-RACE, the RACE-ready complementary DNA (cDNA) was got using SuperScript II (Invitrogen) and the primer GSP1; after synthesizing the cDNA, the deoxycytidine triphosphate and terminal deoxynucleotide transferase were used to add a poly-C tail to the 3′ end of the cDNA; the primer pair of GSP2 and AAP was used to carry out the first PCR, the product of which was reamplified by GSP3 and AUAP according to the same thermal profile, which includes 2 minutes of an initial denaturation at 94°C, then 35 cycles comprising 30 seconds of denaturation at 94°C, 30 seconds of annealing at 55°C, and 2 minutes of extension at 72°C, 7 min of final extension at 72°C, and holding at 4°C at last. For the 3′-RACE, Oligo dT-3 sites Adaptor Primer (Takara) and AMV Reverse Transcriptase XL (Takara) were used to get the RACE-ready cDNA. The temperature profile employed was as follows: 10 minutes of at 30°C, 30 minutes at 50°C, 5 minutes at 95°C, and 5 minutes at 5°C. All of the RACE-ready cDNA products were amplified using sense primers (GSP-3′ RACE) and antisense primers (3 sites Adaptor Primer, Takara), where the temperature profile was as follows: 5 minutes at 95°C; 30 cycles including 30 seconds at 94°C, 30 seconds at 55°C, and 5 minutes at 72°C; and then 7 minutes at 72°C. The PCR products of 5′-RACE and 3′-RACE were purified, ligated into the pMD18-T vector, and sequenced, respectively.

### 2.3. Expression Analysis of the cgCaM Gene in Different Organs

The mRNA levels of the cgCaM gene were measured using the method of Quantitative Real-time PCR. The quantities of cgCaM mRNA in different organs were standardized against that of elongation factor 1 (eLF1) mRNA to compensate for variations in volume of input mRNA. All of the reactions were carried out in a 20 *μ*L volume, which contained 10 *μ*L of SYBR Green Master Mix (Tiangen), 5 pmol of each primer, 7 *μ*L of water, and 1 *μ*L of cDNA obtained by reverse transcription from each tissue (hemolymph, mantle, digestive gland, gill, adductor muscle, and labial palps) RNA using Moloney murine leukemia virus reverse transcriptase (Tiangen, China). Each cDNA sample was amplified with the primers CaM-F and CaM-R in three duplicates, each cDNA sample with the primers eLF1-F and eLF1-R in three duplicates, and the negative control in three duplicates on the same 96-well microplates. The PCR program was used according to the recommendation in the manufacturer. The specificity of the PCR products was evaluated by melting-curve analysis. The data were analyzed using the 2^−ΔΔCt^ method.

### 2.4. Sequence Character Analysis

The online tool, Compute pI/Mw, was used to predict the isoelectric point and molecular weight of cgCaM protein (http://ca.expasy.org/tools/pi_tool.html), and another online tool, the PSORT II program, was used to predict the N-terminal signal and cell location (http://psort.ims.u-tokyo.ac.jp/). Functional domain (IPR) prediction was performed at http://prosite.expasy.org/.

### 2.5. Phylogenetic Tree Construction

The CaM sequences of other mollusks were downloaded from NCBI and the CaM sequence from* Hydroides elegans* was used as an outgroup to root the tree. All the CaM sequences with complete CDS were got by the experimental methods in previous studies and downloaded from NCBI (http://www.ncbi.nlm.nih.gov/). A multiple sequence alignment was obtained using CLUSTALW. Phylogenetic tree was constructed using the neighbor-joining algorithm of MEGA 6 [9]. The robustness of the support for the tree was estimated based on 500 bootstrap replicates. In all of these analyses, the pairwise deletion option was used for handling alignment gaps as missing characters.

### 2.6. Calculation of the Numbers of EF Hand Clan Members in the Genomes of Multiple Species

We downloaded the Alignment file (Stockholm format) of the EF hand clan members from http://pfam.xfam.org/clan/CL0220, converted them into HMM file using hmmbuild tool in HMMER software and searched these motifs in the genomes of* Caenorhabditis elegans*,* Schistosoma ansoni*,* Schistosoma haematobium*,* Capitella teleta*,* Helobdella robusta*,* Culex quinquefasciatus*,* Drosophila melanogaster*,* Octopus bimaculoides*,* Daphnia magna*,* Daphnia pulex*,* Ciona intestinalis*,* Branchiostoma floridae*,* Callorhinchus milii*,* Saccoglossus kowalevskii*,* Aplysia californica*,* Biomphalaria glabrata*,* Pinctada fucata*,* Lingula anatina*,* Acropora digitifera*,* Strongylocentrotus purpuratus*,* Lottia gigantea*,* Homo sapiens*,* Gallus gallus*,* Crassostrea gigas*,* Chrysemys picta*,* Pelodiscus sinensis*,* Lepisosteus oculatus*, and* Salmo salar* using hmmsearch tool and calculated the motif numbers in these genomes. According to the degree of calcification,* Caenorhabditis elegans*,* Schistosoma ansoni*,* Schistosoma haematobium*,* Capitella teleta*,* Helobdella robusta*,* Culex quinquefasciatus*,* Drosophila melanogaster*,* Octopus bimaculoides*,* Daphnia magna* and* Daphnia pulex* were placed into the lowly calcified group;* Ciona intestinalis*,* Branchiostoma floridae*,* Callorhinchus milii*,* Saccoglossus kowalevskii*,* Aplysia californica*,* Biomphalaria glabrata*,* Pinctada fucata*,* Lingula anatina*,* Acropora digitifera*,* Strongylocentrotus purpuratus*, and* Lottia gigantea* were placed into moderately calcified group;* Homo sapiens*,* Gallus gallus*,* Crassostrea gigas*,* Chrysemys picta*,* Pelodiscus sinensis*,* Lepisosteus oculatus,* and* Salmo salar* were placed into highly calcified group.

## 3. Results

### 3.1. Molecular Characteristics of cgCaM

**Figure 1:**
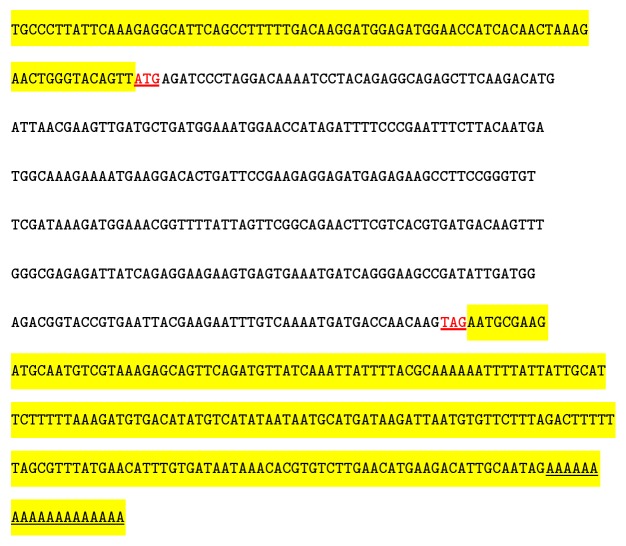
Sequence structure of cgCaM. The sequence highlighted in the upstream region indicates the 5′ UTR and that in the downstream region is the 3′ UTR. “ATG” in bold indicates the initiation codon and “TAG” in bold is the termination codon.

The full-length cgCaM cDNA, which comprised 648 nucleotides, was obtained using the RACE technique. As shown in Figure 1, the ORF comprised 342 nucleotides and it encoded a peptide of 113 amino acids, the 5′ UTR 80, and the 3′ UTR 226 (GenBank accession number KM115543).

The cgCaM peptide was predicted to have an isoelectric point of 4.24 and a molecular weight of 12.86 kDa using the Compute pI/Mw tool. According to the PSORT II program, the cgCaM peptide was predicted to have no N-terminal signal peptide and it was expected to be located in the cytoplasm (probability = 60.9%), which is similar to the CaM and CaLP proteins in* Pinctada fucata* (pfCaM and pfCaLP).

**Figure 2:**
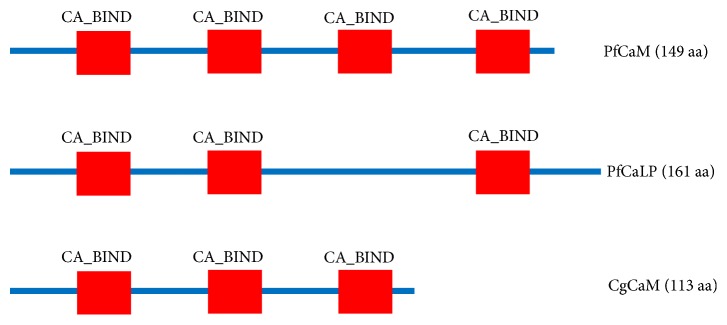
Functional domains of pfCaM, pfCaLP, and cgCaM. The InterProScan domain (IPR) was predicted at http://prosite.expasy.org/. The red block denotes the EF-hand calcium-binding domains (CA_BIND), which were located at amino acids 8–43, 45–80, and 81–113 in the Pacific oyster.

After functional domain prediction, it was found that the cgCaM protein contained three EF-hand calcium-binding domain (CA_BIND) domains at amino acids 8–43, 45–80, and 81–113, and pfCaM also had three CA_BINDs (Figure 2). We did not identify an extra hydrophilic tail specifical for CaLP or any signal peptide in cgCaM [10, 11].

### 3.2. Expression Pattern of the cgCaM Gene in Different Organs

**Figure 3:**
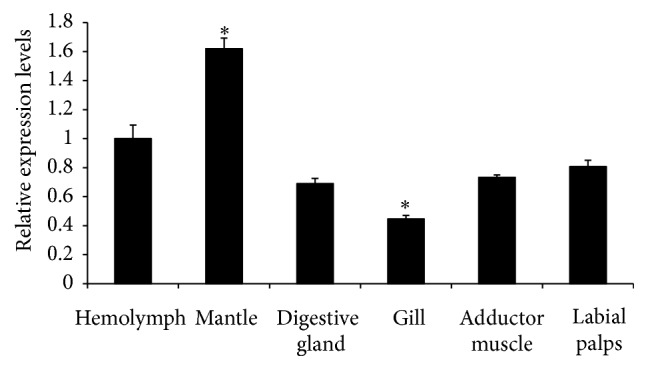
Relative expression levels of the cgCaM gene in different organs of Pacific oyster, that is, hemolymph, mantle, digestive gland, gill, adductor muscle, and labial palps. Asterisk (*∗*) denoted that the cgCaM gene was expressed significantly highly (*p* < 0.05) in the mantle and significantly lowly in the gill (*p* < 0.05).

The mRNA expression levels of the cgCaM gene were examined by real-time PCR and compared indifferent organs (hemolymph, mantle, digestive gland, gill, adductor muscle, and labial palps). As shown in Figure 3, cgCaM was expressed in all six of the tissue types that we investigated, where the cgCaM mRNA expression level was highest in the mantle and lowest in the gill (*p* < 0.05).

### 3.3. Phylogenetic Tree of Peptides Orthologous to CaM

**Figure 4:**
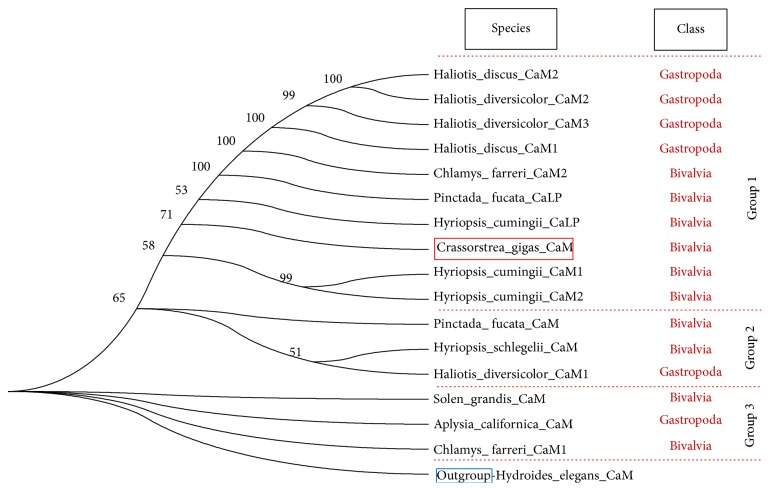
Phylogenetic tree of peptides orthologous to mollusKan CaM. The peptides were divided into 3 groups including group 1, group 2, and group 3. CaM from* Hydroides elegans* was used as an outgroup.

As shown in Figure 4, group 3 appeared earlier than group 2, and group 2 appeared earlier than group 1. Based on the phylogenetic tree of CaM and CaLP, we found that several Gastropoda and Bivalvia CaMs (group 3) were more similar with the outgroup CaM, whereas other molluscan CaMs (groups 1 and 2) were obviously different from the outgroup, while cgCaM appeared in group 1.

### 3.4. The Numbers of EF Hand Clan Members in the Genomes of Multiple Species

**Figure 5:**
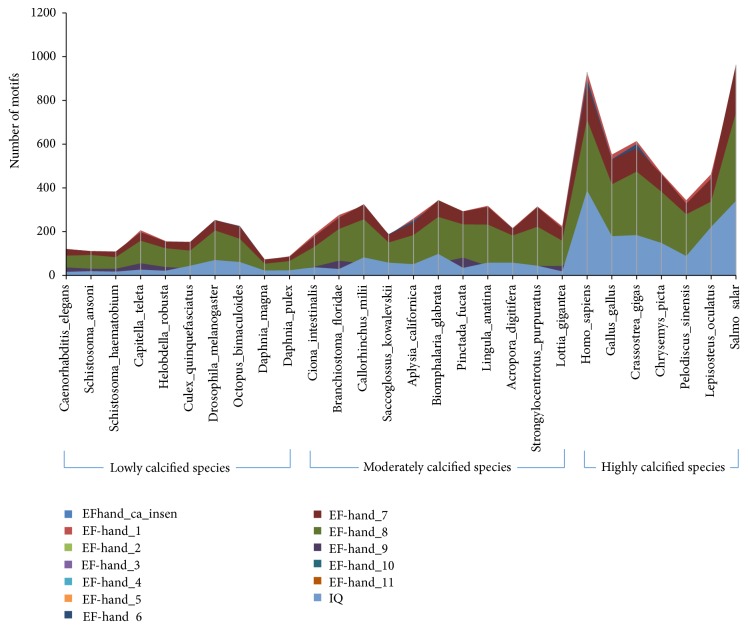
The distribution of EF hand motifs in lowly calcified, moderately calcified, and highly calcified species. EFhand_Ca_insen, EF-hand_1, EF-hand_2, EF-hand_3, EF-hand_4, EF-hand_5, EF-hand_6, EF-hand_7, EF-hand_8, EF-hand_9, EF-hand_10, EF-hand_11, EF-hand_like, and IQ belong to EF hand clan.

**Figure 6:**
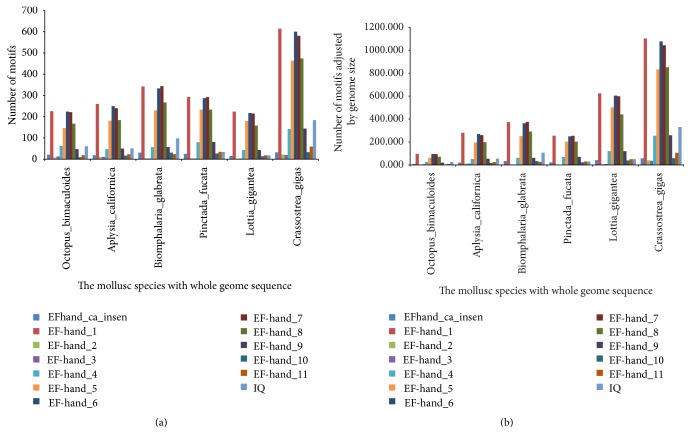
The numbers of some EF hand motifs in whole genomes of some mollusk species. (a) The motif numbers without adjustment; (b) the motif numbers adjusted using genome size.

It was found that the numbers of some EF hand family members, including EFhand_Ca_insen, EF-hand_1, EF-hand_2, EF-hand_3, EF-hand_4, EF-hand_5, EF-hand_6, EF-hand_7, EF-hand_8, EF-hand_9, EF-hand_10, EF-hand_11, EF-hand_like, and IQ, in highly calcified species were higher than those in lowly and moderately calcified species (Figure 5). Interestingly, the numbers of above motifs in oyster genome were the highest among the mollusk species with whole genome sequence (Figure 6).

## 4. Discussion

The shell is one of the most important features of mollusks because it is related to their health and growth. Thus, elucidating the mechanism of shell formation will be beneficial for mollusk culture. PfCaM and pfCaLP are believed to play important roles in shell formation of the pearl [10, 11] oyster and it was concluded that cgCaM may play a different role in shell formation by the Pacific oyster because the composition of the Pacific oyster shell is different from that of the pearl oyster. Determining the sequence characters, expression pattern, and phylogenetic location of cgCaM will facilitate further research into shell formation by the Pacific oyster.

The mantle is an important organ for shell formation [12, 13], although some other organs may also be involved in the process [14, 15]. We found that the cgCaM gene expression level was highest in the mantle, thereby suggesting that its function is probably related to shell formation. However, CaM is present not only in mollusks but also in other animals without shell, such as* Hydroides elegans* [16] (Figure 4), which indicates that CaM has fundamental roles in calcium metabolization but not limited to shell formation and it may be involved in various pathways. Interestingly, we found that Gastropoda and Bivalvia CaMs were present in each branch (group) of the molluskan CaM tree (Figure 4), which suggests that the common ancestor of Gastropoda and Bivalvia probably had at least three CaM genes. In addition, the CaLP genes were only present in group 1 and group 1 was far away from the outgroup, which indicates that the CaLP gene emerged later than the CaM genes.

It was found that highly calcified species had more EF hand clan members in their genomes (Figure 5), hinting the probable correlation between these motifs and biomineralization. As we all know, oyster had the large and thick calcified shells [14, 15]; we also found more EF hand clan members in its genome than in other mollusk genomes (Figure 6), so it could be deduced that more EF hand clan members may promote the formation of large and thick calcified shells. CaM mainly contained three motifs, EF-hand_1, EF-hand_2 and EF-hand_7, and may be involved in shell formation of other mollusks [1–3]; cgCaM also contained these three motifs and expressed highly in mantle (Figure 3), all of which hinted that cgCaM may be involved in oyster shell formation.

## Figures and Tables

**Table 1:** Primers used for CaM cloning and expression analysis.

Primer name	Sequence (5′ → 3′)	Size (nt)	Application
3 sites Adaptor Primer	CTGATCTAGAGGTACCGGATCC	22	3′ RACE
Oligo dT-3 sites Adaptor Primer	CTGATCTAGAGGTACCGGATCC(T)n	22+n	3′ RACE
GSP-3′ RACE	GATGCTGATGGAAATGGAAC	20	3′ RACE
GSP1	GGCTTCTCTCATCTCCTCTT	20	5′ RACE
GSP2	CTTCTCTCATCTCCTCTTCG	19	5′ RACE
GSP3	TTCTCTCATCTCCTCTTCGG	19	5′ RACE
AUAP	GGCCACGCGTCGACTAGTAC	20	5′ RACE
AAP	GGCCACGCGTCGACTAGTACGGGIIGGGIIGGGIIG	38	5′ RACE
CaM-F	GATGCTGATGGAAATGGAAC	20	For CaM gene in real-time PCR
CaM-R	TTCACTCACTTCTTCCTCTG	20	For CaM gene in real-time PCR
eLF1-F	ACCACCCTGGTGAGATCAAG	20	For internal reference gene in real-time PCR
eLF1-R	ACGACGATCGCATTTCTCTT	20	For internal reference gene in real-time PCR
